# Immunoglobulin G4-Related Disease: A Case Report

**DOI:** 10.7759/cureus.31403

**Published:** 2022-11-12

**Authors:** Basavaraj Jatteppanvar, Pradeep Chakravarthy, Mani Teja K, Aditya Sudan, Ravikant .

**Affiliations:** 1 Internal Medicine, All India Institute of Medical Sciences, Rishikesh, IND; 2 Internal Medicine, All India Institute of Medical Sciences, Dehradun, IND

**Keywords:** parotid gland swelling, mikulicz’s disease, sjogrens syndrome, corticosteroids, igg4 -related disease

## Abstract

Immunoglobulin G4-related disease (IgG4 RD) has a fair prognosis but its diagnosis has been difficult due to the condition's wide range of clinical manifestations, limited awareness among common practitioners, and various differentials. Here, we present a case of an elderly male who presented with recurrent dental caries, recurrent sinusitis, persistent dry mouth, and dry eyes along with bilateral parotid gland enlargement without any lymphadenopathy. The patient was evaluated further and found to have elevated levels of IgG4 and on histopathological examination of the parotid gland showed lymphocytic infiltrate with germinal centers without any granulomatous lesions and IgG4-positive plasma cells on immunohistochemistry (IHC). The patient was diagnosed with IgG4 RD and was started on corticosteroids, after which there was a symptomatic improvement.

## Introduction

Immunoglobulin G4-related disease (IgG4 RD) is a recently recognized disease around two decades ago and is a fibro-inflammatory condition characterized by tumefactive lesions, a dense lymphoplasmacytic infiltrate rich in IgG4-positive plasma cells, storiform fibrosis, and often elevated serum IgG4 concentrations [1]. This disorder has a huge spectrum and can impact almost any organ system. Major salivary glands, periorbital tissues, pancreas, biliary tree, kidney, lungs, lymph nodes, and retroperitoneum are frequently involved organs. Mikulicz syndrome (MS), one of the previous names for IgG4 RD, is a rare chronic condition characterized by idiopathic, bilateral, painless, and symmetrical abnormal enlargement of glandular tissue in the head and neck [2]. One of the most typical presentations of IgG4 RD is now recognized to be patients who initially present with enlarged lacrimal and parotid glands. The majority of people who develop this disease are in their middle to late years and the gender distribution varies depending on which organs are affected. However, among the group presenting with salivary gland involvement, the sex ratio may be similar. Immunoglobulin G4-related disease generally affects men more than women [3]. As IgG4 has a good response to medical therapy, early diagnosis and prompt treatment become pivotal. Here, we report a case of IgG4 RD with the classical presentation.

## Case presentation

A 73-year-old man formerly employed as a glass factory manager had a 15-year history of recurrent dental caries for which he has had to replace all of his teeth with artificial dentures as well as a nine-month history of recurrent sinusitis. For five months, the patient had complained of persistent dry mouth as well as a persistent need to drink fluids with meals and associated with recurrent feelings of foreign body sensation in the eyes and dryness without reddish discoloration or blurring of vision (sicca symptoms). This was also associated with gradual onset progressive painless swelling over both sides of the face, and gradual onset persistent feeling of heaviness in the eyelids. No history was suggestive of nasal polyps, asthma, eczema, or other tumefactive lesions that resemble malignancy. He denied any rash, photosensitivity, Raynaud's phenomenon, joint pain, headache, abdominal pain, chest discomfort, or shortness of breath. On examination, a firm, fixed, non-tender bilateral symmetrical enlargement of the parotid gland was noted without any redness or pus discharging sinus, no lymphadenopathy, pallor, clubbing, icterus, and the rest of the general physical and systemic examinations were unremarkable.

**Figure 1:**
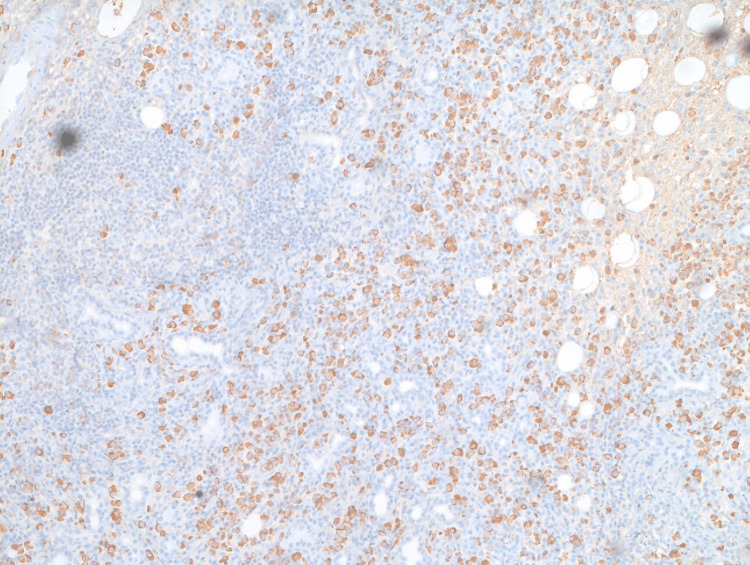
Immunohistochemistry with IgG4 staining showing highlights of IgG4-positive plasma cells IgG4: Immunoglobulin G4

**Figure 2:**
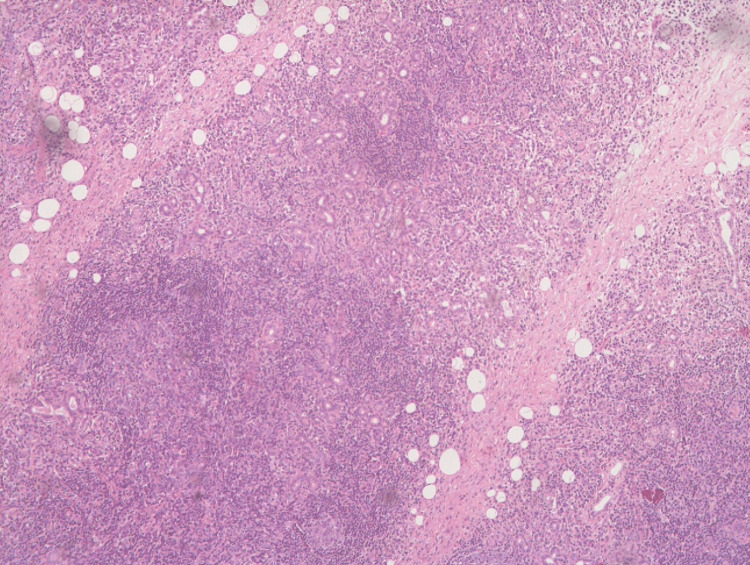
Histopathology examination shows a section of the salivary gland with vaguely maintained lobular architecture and the destruction of acini by lymphoid cell-rich inflammation with the formation of germinal centers. Remnant ducts show mild proliferation and infiltration of lymphocytes forming lymphoepithelial lesions. No significant plasma cells/eosinophils are seen. There is no evidence of granulomatous pathology.

Routine investigations revealed azotemia and mild anemia. Initially, he was worked up along the lines of Sjogren's syndrome as the patient is having significant xerostomia, and xeropthalmia after ruling out diabetes, HIV, mumps, alcoholism, hypothyroidism, and heavy metal exposure. The ophthalmological evaluation was done for sicca symptoms and was not satisfactory with Sjogren's syndrome. His antinuclear antibodies (ANA)-immunofluorescence assay (IFA) came to be negative, Sjögren's-syndrome antigen A (SSA) antibody low titer positive, low C3, normal C4, and angiotensin-converting enzyme (ACE) levels within normal limits. With high suspicion index of IgG4 RD, he was further evaluated for IgG4 levels which were found to be elevated. As per the European League Against Rheumatism (EULAR)/American College of Rheumatology (ACR) Classification and Diagnostic criteria for IgG4 RD, a workup was done. Immunohistochemistry (IHC) showed highlights of IgG4-positive plasma cells (Figure 1). Histopathological examination of the parotid gland showed lymphocytic infiltrate with germinal centers without any granulomatous lesion (Figure 2). As per ACR/EULAR criteria, contrast-enhanced computed tomography (CECT) of the thorax and abdomen was done which revealed multiple hypo-enhancing lesions involving both the kidneys and multiple small focal outpouchings involving the sigmoid colon, likely diverticulosis. Few round soft-tissue attenuations in the upper lobes of bilateral lungs with mediastinal lymphadenopathy and asymmetrical thickening involving the pyloric canal of the stomach for a length of 3.8cm and width of 1.3cm with the loss of mural stratification. Considering the relatively increased risk of gastric carcinoma in patients with IgG4-related disease, upper gastrointestinal endoscopy and biopsy were done which showed no evidence of dysplasia/malignancy. 

## Discussion

There are many close differential diagnoses for IgG4 RD. The clinical presentation of sarcoidosis matches this disease. In our patient, after ruling out the main causes for sicca symptoms and bilateral symmetric painless parotid enlargement, Sjogren’s syndrome is of great interest in our case and SSA low titer positivity further makes Sjogren’s a very close differential here [4]. Immunoglobulin G4-related disease typically manifests in the sixth decade of life, with persistent glandular growth, low prevalence of ANA (30%), high levels of IgG4 and IgG4/IgG index, seronegative for anti-Ro and anti-La (SSA, SSB), advanced storiform fibrosis (from the center to the periphery), glandular preservation, venular obliteration (obliterative phlebitis), and excellent response to steroids. Sjogren's is more prevalent in women in their fifth decade of life with more xerophthalmia and xerostomia, ANA (90%), and anti-SSA (50%) with the ability to progress to glandular destruction and be resistant to steroids [5]. Immunoglobulin G4-related disease can cause serious organ dysfunction and failure [6,7,8]. There are a few case reports in the literature about the various clinical presentations of this disease since 2000. But there was confusion in diagnosis as we did not have standardized diagnostic and classification criteria with good sensitivity and specificity. In our study, we applied the classification criteria recommended by ACR/EULAR for IGG4 RD which consists of three steps [9].

**Figure 3:**
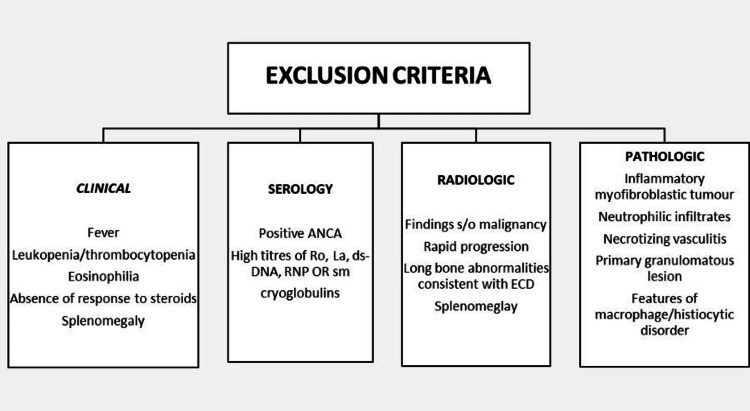
Exclusion criteria ANCA: Antineutrophil cytoplasmic antibodies, RNP: Ribonucleoproteins, ECD: Erdheim–Chester disease

The first step is entry criteria which need a demonstration of IgG4 RD manifestation in at least one of 11 possible organs in a manner consistent with this disease; our patient has characteristic ocular and major salivary glandular enlargement and on histopathology with IHC has a picture consistent with IgG4 RD. The second step is excluding the other common misdiagnosis made with this disease by using the following criteria which have been divided into clinical, serological, radiological, and pathological features that should not be present to make a diagnosis of IgG4 RD (Figure 3).

Our patient did not have any systemic inflammatory features; on serology, he had SSA (Ro) positive but in low positive titer, and on CECT abdomen, he had pyloric thickening which made gastric malignancy a possibility. Hence an upper GI endoscopy with a biopsy was done which ruled out malignancy. In histopathology, there were no features elucidated above. The third step is the application of weighted inclusion criteria based on clinical, serological, radiological, and pathological assessments with scoring.

The IgG4 RD classification criteria are fulfilled if entry criteria are met, no exclusion criteria are present, and the total points add up to 34 for our patient ( ≥20) (Table 1). The new IgG4-RD criteria, according to the researchers are not meant to be applied in clinical settings to make diagnosis. Failure to meet all classification criteria when a patient is clinically diagnosed with IgG4 RD should not prevent disease management, but the criteria may offer a helpful framework for clinicians to understand an IgG4-RD diagnosis in a patient and highlight findings that raise the likelihood that a patient has IgG4 RD. The exclusion criteria also shouldn't be viewed as a list of investigations or tests, clinicians must perform on every patient and corticosteroids remain the most beneficial treatment in IgG4-related disease [10]. Similarly, our patient had a regression in the size of the bilateral parotid gland and symptomatic relief following the corticosteroid treatment. 

**Table 1:** IgG4-related disease classification criteria IgG4: Immunoglobulin G4

1)	Histopathology: Dense lymphocytic infiltrates immunostaining	+4
2)	Serum IgG4 concentration – 3 x Upper normal limit	+6
3)	3 Gland involvement – Parotid, Submandibular, Lacrimal	+14
4)	Chest	0
5)	Pancreas and Biliary tree	0
6)	Kidney – Bilateral renal cortex hypodensities	+10
7)	Retroperitoneum	0
	Total Inclusion points	34

## Conclusions

Immunoglobulin G4-related disease is a clinically evident condition that typically manifests sub-acutely but it can occasionally progress over several months, years, or even decades. Many individuals with IgG4 RD are misdiagnosed as having other diseases, including malignancies because of their findings, which can be attributed to non-specific inflammation. This hinders early initiation of treatment and reduces the patient's quality of life. Many patients with the disease develop an indolent form of the disease that remains asymptomatic for years without progressing to other disease manifestations; therefore, not all disease manifestations require prompt medical attention; in some cases, watchful waiting is recommended, but vital organ involvement must be treated aggressively. However, IgG4 RD can cause serious organ dysfunction and failure. Steroids are still the first line of treatment for IgG4 RD patients because they are efficacious, particularly when started early in the disease's course. In some cases, a combination of immunosuppressive agents is required. 
